# Reticulation of Block Copolymer Nanostructures from Perforation

**DOI:** 10.1021/acsami.4c20386

**Published:** 2025-02-12

**Authors:** Shih-Lin Yeh, Cheng-Yen Chang, Rong-Ming Ho

**Affiliations:** Department of Chemical Engineering, National Tsing Hua University, Hsinchu 30013, Taiwan, R.O.C.

**Keywords:** PS-*b*-PDMS, controlled self-assembly, epitaxial growth, hexagonally perforated lamellae, double gyroid, double diamond

## Abstract

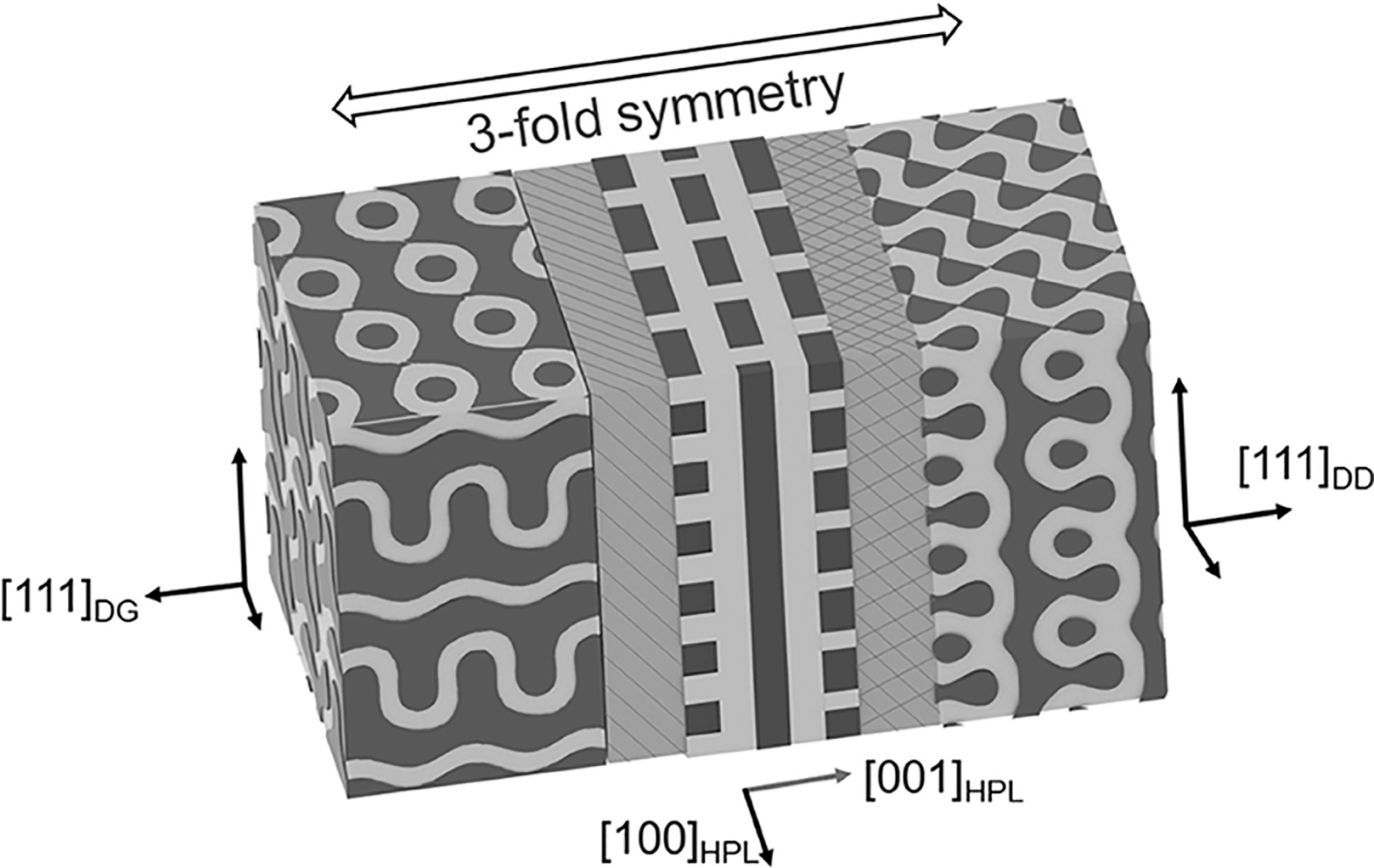

This work aims to examine a variety of metastable phases from the controlled self-assembly of a lamellae-forming polystyrene-*block*-polydimethylsiloxane (PS-*b*-PDMS) and its blends with a PDMS homopolymer. Kinetically trapped phases including hexagonally perforated lamellae (HPL), double diamond (DD), and double gyroid (DG) can be obtained from the blends, making it feasible to investigate the transition mechanisms from perforation to reticulation for the formation of network phases (i.e., DD and DG) as evidenced by temperature-resolved small-angle X-ray scattering experiments. Most interestingly, on the basis of the 3D reconstruction of transmission electron microscopy (TEM) images (electron tomography), an epitaxial relationship between the [001] direction of HPL and the [111] direction for DG and DD phases for the transformations from HPL to DG and DD, respectively, can be clearly identified. Specifically, the 3D double networks of PDMS are initiated from the parallel PDMS layers with PS perforation, forming the topological building units for the gyroid (trigonal planar texture) and diamond (tetrapod texture) phases. As a result, this finding may fill up the lost parts of the morphological evolution from perforation to reticulation in terms of topological transformations.

## Introduction

In recent years, fascinating well-ordered network structures found in natural species and synthetic materials have been appealing due to their deliberate structuring for superior properties.^1−8^ Inspired by nature, the self-assembly of block copolymers (BCPs) has been extensively studied because of the formation of a wide variety of nanonetwork phases, in particular, gyroid (G) and diamond (D).^9−15^ Apart from the bicontinuous nanostructure, a unique complex phase with a perforated structure is also observed. In contrast to the unperforated lamellar phase with a continuous texture from each component, the perforated structure is formed by hexagonally packed channels of the majority component material extending through the minority component layers to form a monocontinuous structure, and therefore, it is named the hexagonally perforated lamellae (HPL) phase.^16−22^ Specifically, the special perforated structure presents a combination of 2D and 3D continuity with distinct rheological responses.^16^ Moreover, the perforated structure is able to create continuous and regulated pathways for the transportation of fluids after the removal of degradable blocks,^7,23,24^ which exhibits great potential as a mesoporous membrane with uniform pore sizes for ultrafiltration.^25−31^ Even though the HPL phase is considered an isotropic structure, the high morphology factor of the perforation (2D and 3D continuity) allows greater flexibility of the control of structural orientations for high permeability. Because of the fascinating structural characteristics, the stacking sequence can be modeled as AB and ABC-type in a hexagonally packed symmetry.^20^ In common phase diagrams of diblock copolymers, a narrow composition window of the HPL phase was discovered and has been well studied extensively.^32,33^ However, the HPL structure is regarded as a metastable phase as compared to the double gyroid (DG) phase in a diblock system, and therefore, the phase transition from HPL to DG has been comprehensively studied experimentally and theoretically.^19,32,34−38^ An epitaxial relationship between the HPL layers and the DG phase along the [211]_DG_ direction was clearly observed^19,36,37^; According to the current findings, discoveries of epitaxial growth from the HPL to the DG phase were used from the specific shear-aligned HPL phase in most of the studies. Few reports reveal the occurrence of HPL–DG transitions under confinement in the film state.^39−41^ More results of visualization of the transition have been reported by Chang and co-workers.^42,43^ Based on previous reports, the forming windows for the network phases are usually narrow, and the window of the metastable HPL phase appears in the neighbor;^32,33^ how to enlarge the window or efficiently induce a network formation (i.e., reticulation) is essential. Furthermore, in a previous study, it has been proven that a metastable double diamond (DD) phase and a DG phase can be kinetically captured by the controlled self-assembly of single-composition polystyrene-*block*-polydimethylsiloxane (PS-*b*-PDMS) using a PS-selective solvent for casting.^44,45^ By controlling the evaporation rate of the PS-selective solvent, both DD and DG phases can be acquired.^45^ Owing to the similar space symmetry among the DD, DG, and HPL phases, which share the same 3-fold rotational symmetry and similar compositions, an order–order transition can be expected.^46,47^ In other words, the networking into a bicontinuous structure may be developed from a monocontinuous structure with perforation. Especially for the study of the HPL–DD transitions, this has never been revealed.

In this study, different self-assembled morphologies can be acquired from single-composition lamellae-forming PS-*b*-PDMS by using different selective solvents. A facile method proposed to acquire diverse morphologies was by using a selective solvent for network formation from lamella-forming PS-*b*-PDMS and also by mixing with the low-molecular-weight PDMS homopolymer. Interestingly, a kinetically trapped HPL phase can be formed by controlling the evaporation rate for solution-casting. More interestingly, it is feasible to acquire network phases through a solid–solid transition from the HPL. Note that the perforation in the HPL can be referred to as an initial stage for the reticulation from two dimensions to three dimensions, thus giving the opportunity for the examination of the reticulation of the gyroid and diamond from perforation. The expected transitions can be evidenced by the small-angle X-ray scattering (SAXS) results, showing the formation of mixing phases with the HPL and network phases. By taking advantage of electron tomography (i.e., three-dimensional transmission electron microscopy (3D TEM)), the reconstruction images allow one to examine the morphological evolution from perforation to reticulation. As found from the tomographic results, the transformation from HPL to DG and DD both adopt “soft” epitaxial relationships along specific directions (Figure S1). Unlike the phase transitions in the atomic level, “hard” epitaxy with sharp and clear boundaries can be identified; the phase transitions of the ordered phases in self-assembled BCPs (i.e., the “soft” matter) undergo a continuous transformation from one phase to the other where the boundaries of the two observed phases turn out as areas (referred to as the transition zone) showing continuous transitions. As a result, these discoveries may provide insights into the epitaxial growth of soft matters with complicated phase behaviors.

## Experimental Section

### Syntheses and Characterization of PS-*b*-PDMS and the PDMS Homopolymer

The synthesis of the PS-*b*-PDMS diblock copolymer was accomplished through sequential anionic polymerization of styrene and hexamethylcyclotrisiloxane (D_3_), employing high-vacuum techniques. More details of the characterization (Figure S2) and the synthetic procedures can be found in a previous report.^45^ Commercial PDMS–OH (Polymer Source Inc.) was used in this study. As illustrated in Figure S3, ^1^H NMR (CDCl_3_): 5.05 (m, 1H, CH), 3.51 (d, 2H, CH_2_), 1.52 (br, 2H, CH_2_), 1.48 (br, 2H, CH_2_), 1.35 (br, 3H, CH_2_), 0.89 (t, 3H, CH_3_), 0.52 (t, 3H, CH_2_), 0.14–0.34 (m, 6H, Si(CH_3_)_2_) can be clearly identified, which is in line with the chemical structure of commercial PDMS–OH. The peak area ratio of peak h (repeating unit) and peak g (*n*-butyl end group) could be calculated to acquire the molecular weight of PDMS–OH. On the basis of the area ratio calculation, the molecular weight of commercial PDMS–OH was determined as approximately 10,000 g/mol, with a low polydispersity at approximately 1.07 (Figure S4). Detailed characterizations of the studied samples are summarized in Table S1.

### Sample Preparation

PS-selective solvent (i.e., chloroform, 9.3 cal^0.5^/cm^1.5^) and neutral solvent (i.e., cyclohexane, 8.2 cal^0.5^/cm^1.5^) with different evaporation rates were used for solution-casting for the PS-*b*-PDMS and PS-*b*-PDMS/PDMS homopolymer blends. The concentrations for all PS-*b*-PDMS or PS-*b*-PDMS/PDMS blend solutions were fixed at a concentration of 10 wt %. The initial amounts of polymer solution were all fixed at approximately 500 mg and kept in cylindrical glass vials (inner diameter ∼7 mm). Different sizes of slits were pinched on the vial caps to control the evaporation rates. Different evaporation rates to the polymer solution were carried out for the study of the controlled self-assembly: slow (0.04 mL/day) and fast (1.2 mL/day) evaporation rates. After drying the samples in ambient conditions for hours or days, the bulk sample was moved to a vacuum desiccator for 1 day to remove the solvent completely.

### Morphological Observation

Ultrathin microsections (thickness lower than 60 nm) of the solution-cast and thermally annealed PS-*b*-PDMS were prepared at −160 °C by a Leica EM UC6 microtome with a Cryochamber EM FC7. Real-spacing images (TEM) were acquired from the ultrathin microsections without staining due to the intrinsic mass–thickness contrast from PDMS to PS microdomains. TEM studies were performed on a JEOL-2100 transmission electron microscope operating at an accelerating voltage of 200 kV.

### SAXS Experiments

The synchrotron beam source from beamline BL23A of the National Synchrotron Radiations Research Center (NSRRC) was used for the SAXS experiments with a mirror germanium (111) double-crystal monochromator was used to vertically focus the incident X-ray beam at the energy of 10 keV. The wavelength of the X-ray beam was 1.24 Å. The beam stop was a round tantalum disk of 4 mm diameter. A MAR CCD X-ray detector (MAR USA) was used to collect the two-dimensional (2D) SAXS patterns. For common measurements at ambient conditions, the solution-cast samples were detached from the glass vials and then transferred to stainless-steel washers for SAXS experiments (see Figure S5 for a detailed illustration). Kapton tapes were used to fix the sample positions in the washers. For temperature-resolved SAXS experiments, the samples were sealed in stainless-steel washers by the Kapton tape under nitrogen to reduce the oxygen content. Temperature-resolved SAXS experiments were carried out using step heating from 30 to 230 °C for all samples after solution-casting. The corresponding SAXS profiles were taken every 5 min.

## Results and Discussion

**Figure 1:**
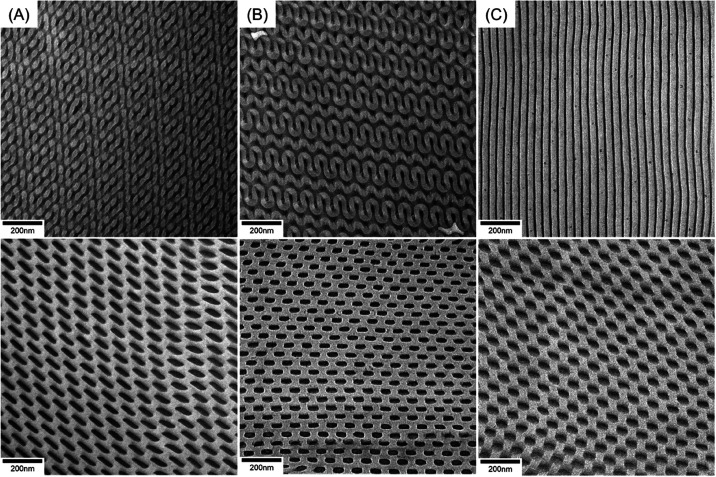
TEM micrographs of PS-*b*-PDMS/PDMS blends with different amounts of PDMS homopolymer of (A) 1.5, (B) 3, and (C) 5 wt %. The self-assembled PS-*b*-PDMS/PDMS blends were prepared by solution-casting using chloroform at different evaporation rates. The upper part shows the ones prepared by solution-casting under a low evaporation rate (0.04 mL/day) of the solvent and the lower part shows those obtained by solution-casting under a high evaporation rate (1.2 mL/day).

**Figure 2:**
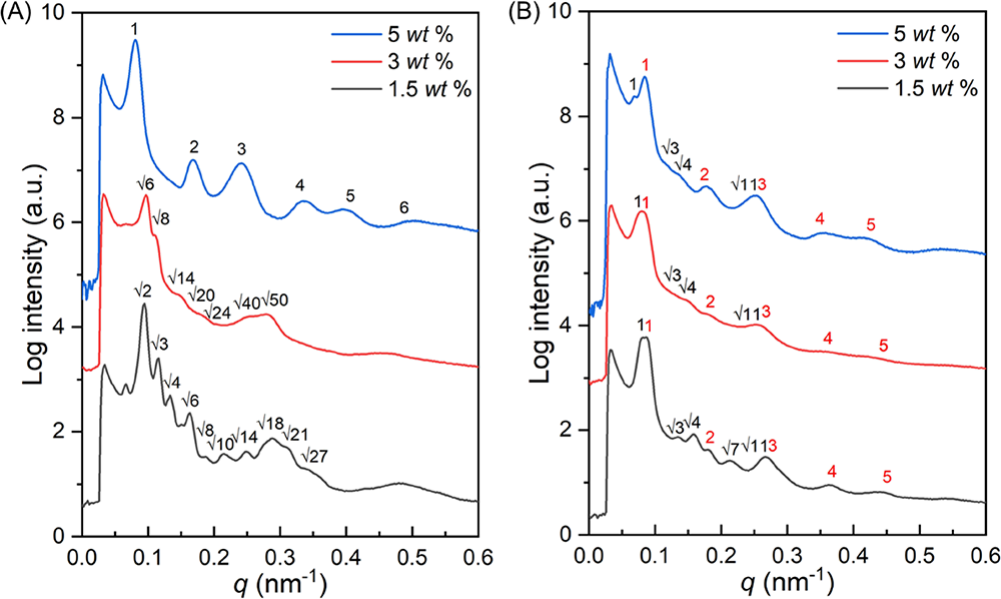
1D SAXS profiles of PS-*b*-PDMS/PDMS blends with different amounts of the PDMS homopolymer after (A) slow (0.04 mL/day) and (B) fast (1.2 mL/day) evaporation for solution-casting from chloroform solution. The intensities of the scattering profiles are shifted vertically by an arbitrary factor to avoid the overlap of peaks.

The equilibrium morphology of lamellae-forming PS-*b*-PDMS with a *f*_PDMS_*^v^* at 0.42 was acquired by using a neutral solvent, cyclohexane, for solution-casting. As shown in Figure S6A, a high mass contrast of silicon-containing PDMS appears as a dark striped microdomain, whereas PS appears as a bright striped microdomain under TEM imaging, reflecting the formation of the lamellar texture after solution-casting. As further evidenced by the SAXS results (Figure S6B), reflections occurring at relative *q* values at 1:2:3:4 can be clearly identified. To create the reticulation of self-assembled morphologies, a PS-selective solvent, chloroform, with a controlled evaporation rate for solution-casting is used, with which it is possible to kinetically capture the metastable network phases. As shown in Figure 1A, with the introduction of a small amount of the PDMS homopolymer (1.5 wt %), the projection along [311] of the DD phase can be observed. The DD-structured blends can be further evidenced by the 1D SAXS profile (Figure 2A) at which the characteristic reflections with relative *q* values of √2: √3: √4: √6: √8: √10: √14: √18: √21: √27 can be identified. Note that chloroform appears to be a strong PS-selective solvent that might significantly reduce the effective volume fraction (*f*_PDMS_^eff^), whereas the introduction of the PDMS homopolymer for blending might alleviate the packing frustration to allow the formation of the DD phase with high packing frustration. Notably, with 3D network characteristics, the *d*-spacing of (110)_DD_ is calculated to be approximately 66.4 nm based on the primary peak. Interestingly, with a slightly larger amount of the PDMS homopolymer added (3 wt %), the projection plane along [211] of the DG phase can be found (Figure 1B), as further evidenced by the SAXS results (Figure 2A) with the reflections at relative *q* values of √6: √8: √14: √20: √24: √40: √50. The results suggest that, with an increase in the PDMS volume fraction, it is possible for the DG phase to form, in line with the expected phase behaviors.^48^ The interdomain spacing (*d*) of (211)_DG_ was calculated to be approximately 64.9 nm from the primary peak of diffraction. Notably, as shown in Figure 1C, with the introduction of a large amount of the PDMS homopolymer into the PS-*b*-PDMS (approximately 5 wt %), alternating PDMS dark stripes and PS bright stripes can be clearly observed by TEM, revealing the formation of a lamellae texture, in line with the reflections occurring at relative values of 1:2:3:4:5:6 in the 1D SAXS profile (Figure 2A). Notably, it is reasonable to expect phase transitions from DD to DG and further to lamellae with an increase in the PDMS homopolymer in the blends. With homogeneous blending after solution-casting, it is feasible to tune the volume fraction of the constituted compositions in the blends, forming the kinetically trapped metastable phases as expected.

In contrast to the slow evaporation, a fast evaporation (1.2 mL/day) was carried out for solution-casting of the blends to acquire a wider variety of metastable phases from kinetically controlled self-assembly. Interestingly, as shown in the lower part of Figure 1, while the amount of introduced PDMS homopolymer is in the range of 1.5–5 wt %, a ladder-like texture, which is presumed to be one of the projections from the HPL phase, from blending can be clearly identified by TEM after solution-casting at a high evaporation rate. All of the HPL-structured blends can be further evidenced by the 1D SAXS profile (Figure 2B) at which two sets of the reflections with relative *q* values of 1: √3: √4: √7: √11 and 1:2:3:4:5 can be identified. Namely, the combined scattering reflections indeed suggest the formation of the HPL phase with hexagonally packed PS columns and alternating perforated layers. Detailed structural information on the self-assembled PS-*b*-PDMS/PDMS blends prepared by solution-casting under different evaporation rates is summarized in Table S2. The addition of the PDMS homopolymer not only alters the effective volume fraction but also alleviates the packing frustration to the metastable phases; in other words, the HPL phase is believed to possess higher entropic penalty.^49^ As a result, the formation of the HPL phase from PS-*b*-PDMS/PDMS blends may provide the opportunity to capture the perforated phases before morphological evolution into long-living network phases.

**Figure 3:**
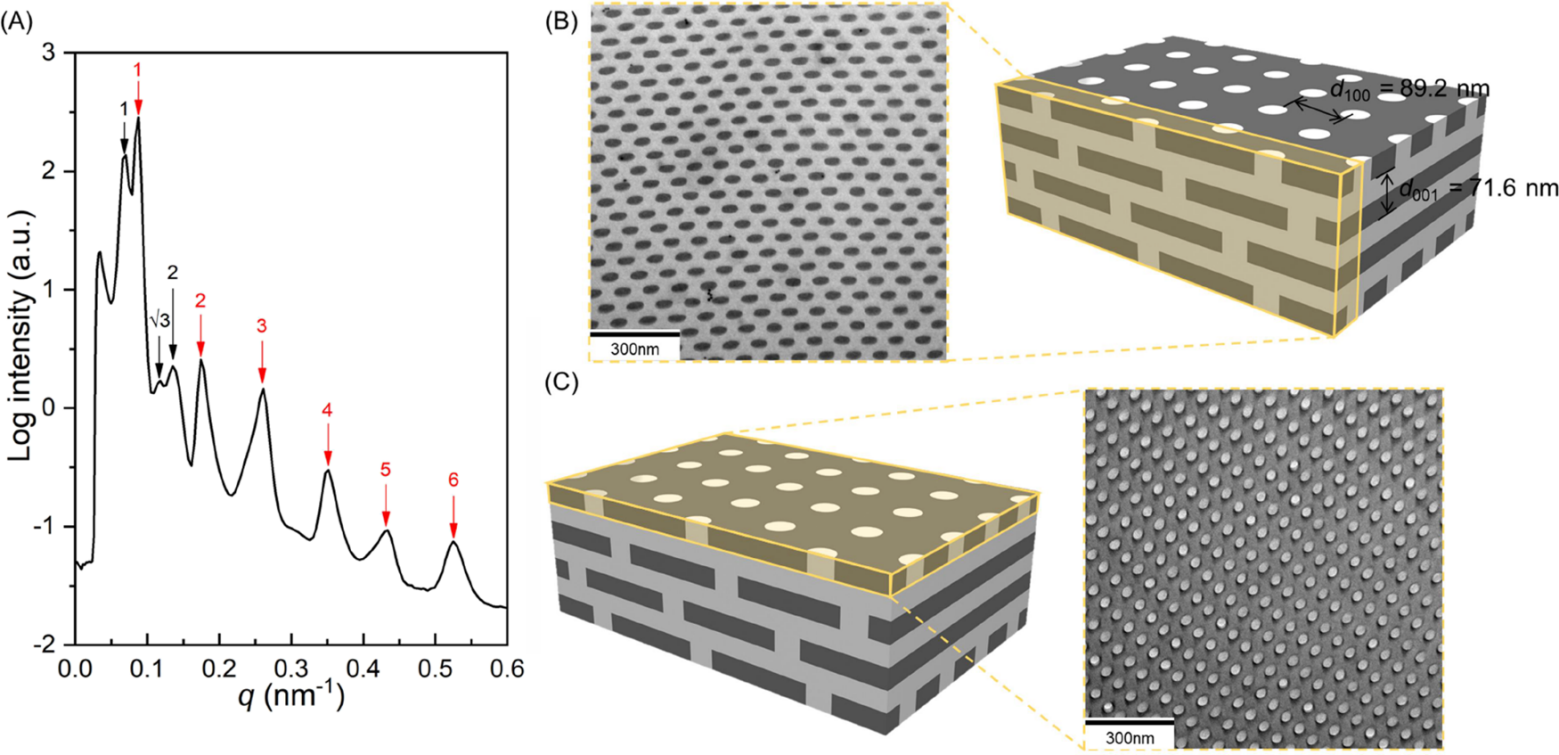
(A) 1D SAXS profile and TEM micrographs of the self-assembled PS-*b*-PDMS/PDMS blends (3 wt % PDMS homopolymer) and the corresponding projection planes viewed from (B) the cross-section and (C) top views of the HPL. Ideal models of the ABC-type stacking HPL phase are illustrated. The PDMS and PS microdomains in HPL are in black and gray colors, respectively.

To further examine the forming HPL phase, it is necessary to understand the forming mechanisms and corresponding transitions for the perforated phase from the intrinsic stable phase (lamellae-forming PS-*b*-PDMS). As reported by Zhou et al., the embryo to HPL should be in a disorder phase but with a ladder-like texture.^50^ Note that there are two different packings with respect to perforated cylinders; they are AB- and ABC-type stackings with *P6*_*3*_*/mmc* and *R*3̅*m* space groups, respectively. As a result, to decipher the intrinsic layered structure and the corresponding stacking from the ladder-like texture, thermal annealing will be carried out to achieve the formation of the HPL phase with high ordering. After thermal annealing at 120 °C above the *T*_g_ of PS (*T*_g_^PS^ ∼ 100 °C) for 10 days, long-range ordering of the HPL phase can be achieved, as evidenced by SAXS results (Figure 3A) at which significant reflections with both lamellar and hexagonally packed characters can be clearly identified. Figure 3B shows TEM projection with the PS perforation through PDMS and layer-by-layer PS at which the mass contrast distinguishes between the dark PDMS from the bright PS. Also, as shown in Figure 3C, a hexagonally packed bright spherical texture (PS microdomain) can be observed by TEM, resulting from the projection viewing from the top of the HPL phase. These results thus evidence the formation of an HPL phase with long-range order. Accordingly, the layer-by-layer PS exhibits a *d*-spacing of 71.6 nm (i.e., *d*_(001)HPL_), whereas the perforated PS exhibits a hexagonal lattice of approximately 89.2 nm (i.e., *d*_(100)HPL_). To further examine the suggested HPL phase, three-dimensional tomography was carried out for the reconstruction of the projection images. As shown in Figure S7, the projection plane based on the reconstructed structure from the specific area marked by a red dashed line in Figure S7A can be examined, at which the dark microdomains represent PDMS, whereas the gray areas represent the PS microdomain. Note that PDMS microdomains perforated with the PS microdomain can be found, leading to the formation of the PS perforation texture from the continuous PDMS layer into the isolated elliptical texture. Moreover, to decipher the corresponding stacking sequence of the forming HPL phase, the reconstruction image was slightly tilted (Figure S7B) for visual clarification. The positions of the perforations can now be clearly observed. As shown by the red lines and alphabetical letters marked in Figure S7B, the perforations from the first layer reappear at the same position in the fourth layer (i.e., marked by letter A). As a result, the position for the PS perforations would be repeated every three layers, indicating an ABC-type stacking with the *R*3̅*m* space group rather than an AB-type stacking with the *P6*_*3*_*/mmc* space group to form the HPL phase.

### Order–Order Phase Transition from HPL to DG

**Figure 4:**
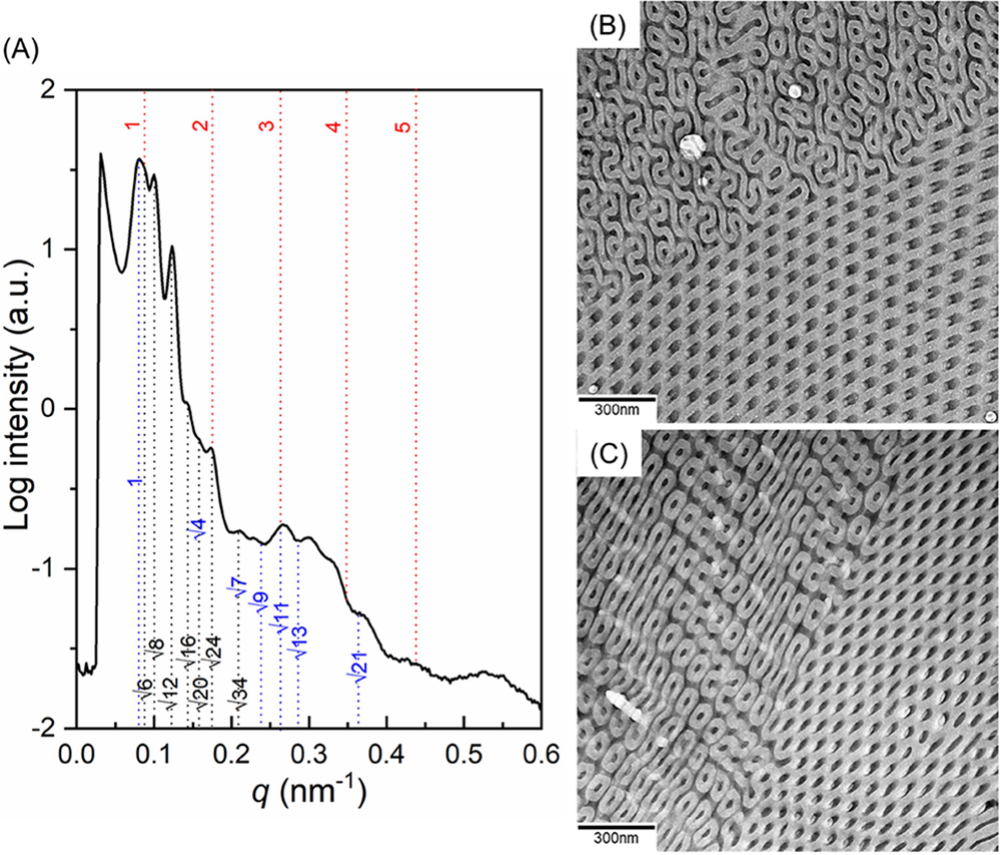
(A) 1D SAXS profile of the solution-cast PS-*b*-PDMS/PDMS blends with 3 wt % PDMS homopolymer after moderate evaporation (0.1 mL/day). The blue and red dashed lines denote reflections corresponding to HPL, whereas the black dashed lines denote reflections from DG. (B, C) Corresponding TEM micrographs of the boundaries between HPL and DG phases from different locations.

**Figure 5:**
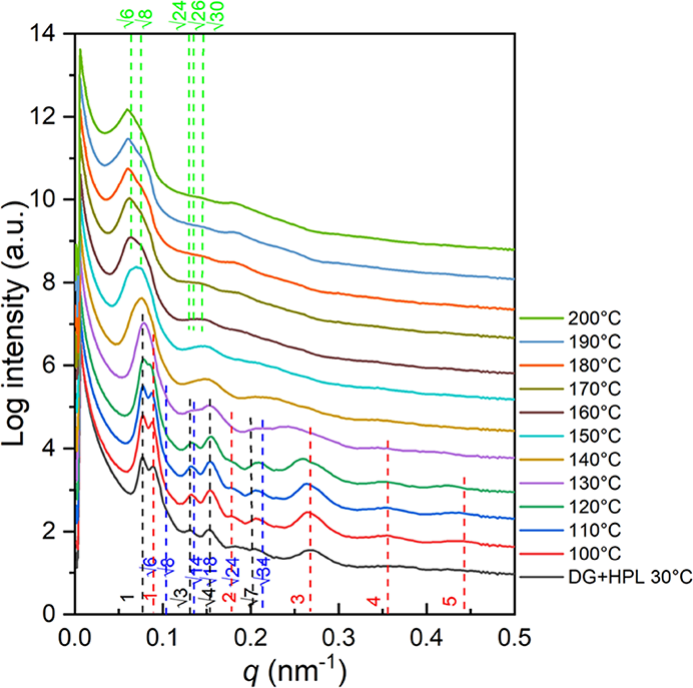
Temperature-resolved in situ 1D SAXS profiles of PS-*b*-PDMS/PDMS blends with 3 wt % PDMS homopolymer after moderate evaporation (0.1 mL/day) for solution-casting. The black and red dashed lines refer to the reflection planes of the HPL phase, whereas the blue and green dashed lines are the reflection planes of the DG phase. The intensities of the scattering profiles are shifted vertically by an arbitrary factor to avoid overlapping of peaks.

Regarding the examined phase behaviors of PS-*b*-PDMS/PDMS blends, systematic studies by controlling the evaporation rate for solution-casting of the blending systems were carried out to clearly examine the transitions from perforation to reticulation. The coexistence of HPL and DG phases can be found in the self-assembled PS-*b*-PDMS/PDMS blends (3 wt % of the homopolymer) after solution-casting under a moderate evaporation rate (0.1 mL/day) in between the formation of the HPL phase at 1.2 mL/day and DG phases at 0.04 mL/day. As shown in Figure 4A, the reflection peaks for the HPL phase are denoted with black dashed lines with relative *q* values of 1: √3: √4: √7: √9: √11: √13: √21 and red dashed lines with relative *q* values of 1:2:3:4:5, whereas the blue dashed lines in the SAXS profile indicate the predicted characteristic reflections of the DG phase occurring at relative *q* values of √6: √8: √12: √16: √20: √24: √34. The 1D SAXS profile indeed shows the reflections from both phases of HPL and DG, suggesting the coexistence of HPL and DG phases. Interestingly, as shown in Figure S8, the lattice of DG (*d*_(211)DG_ ∼ 70.0 nm) acquired from solution-casting under a moderate evaporation rate was calculated to be slightly higher than the one from slow evaporation (*d*_(211)DG_ ∼ 64.9 nm) while the periodicity for HPL (*d*_(100)HPL_) still remains. Furthermore, to investigate the thermodynamic stabilities of the forming coexistent phases, temperature-resolved in situ SAXS experiments were performed. As shown in Figure 5, characteristic reflections from both HPL (black and red dashed lines) and DG phases (blue dashed lines) can be clearly observed. In the first stage (<130 °C), the increase in the intensities of all reflection peaks from both HPL and DG phases can be found due to the ongoing thermal annealing, which causes long-range ordering as the PS matrix devitrifies. Once the temperature reaches 130 °C, reflections from (001)_HPL_ and (100)_HPL_ as well as (211)_DG_ broaden and merge with each other simultaneously, indicating the ongoing order–order transitions from mixed phases into a single phase. Intermediates between the HPL and DG phases were formed, which appear as a bridge (i.e., transition zone) between those two phases. Similar scenarios were observed in the phase transitions among the self-assembled network phases.^45^ When the temperature reaches 140 °C, primary reflection peaks for the HPL phase ((001)_HPL_ and (100)_HPL_) gradually vanish. Consequently, as the temperature increases over 160 °C, the completion of the phase transformation results in a significant reflection at the low-*q* region, suggesting the formation of the DG phase with the reflections from (211)_DG_ and (220)_DG_. The characteristic reflection planes for DG are labeled with green dashed lines at relative *q* values of √6: √8: √24: √26: √30. As a result, the phase transition from HPL/DG mixed phases to the pure DG phase could be observed after high-temperature annealing for a long time, suggesting that the HPL phase should be a metastable phase with lower thermodynamic stability than the DG phase. Also, it reasonably indicates that the metastable HPL phase or coexistent phase can be kinetically trapped by controlling the evaporation rate during solution-casting. Namely, giving enough mobility and relaxation time (i.e., slow evaporation), polymer chains are able to self-assemble into the DG phase with higher thermodynamic stability through a kinetic process. In contrast, a shorter relaxation time (i.e., fast evaporation) will rapidly freeze the polymer chains, resulting in the formation of a metastable HPL phase with lower thermodynamic stability. The transition stage of coexistent HPL and DG phases will be successfully captured once the evaporation rate is precisely controlled during solution-casting.

**Figure 6:**
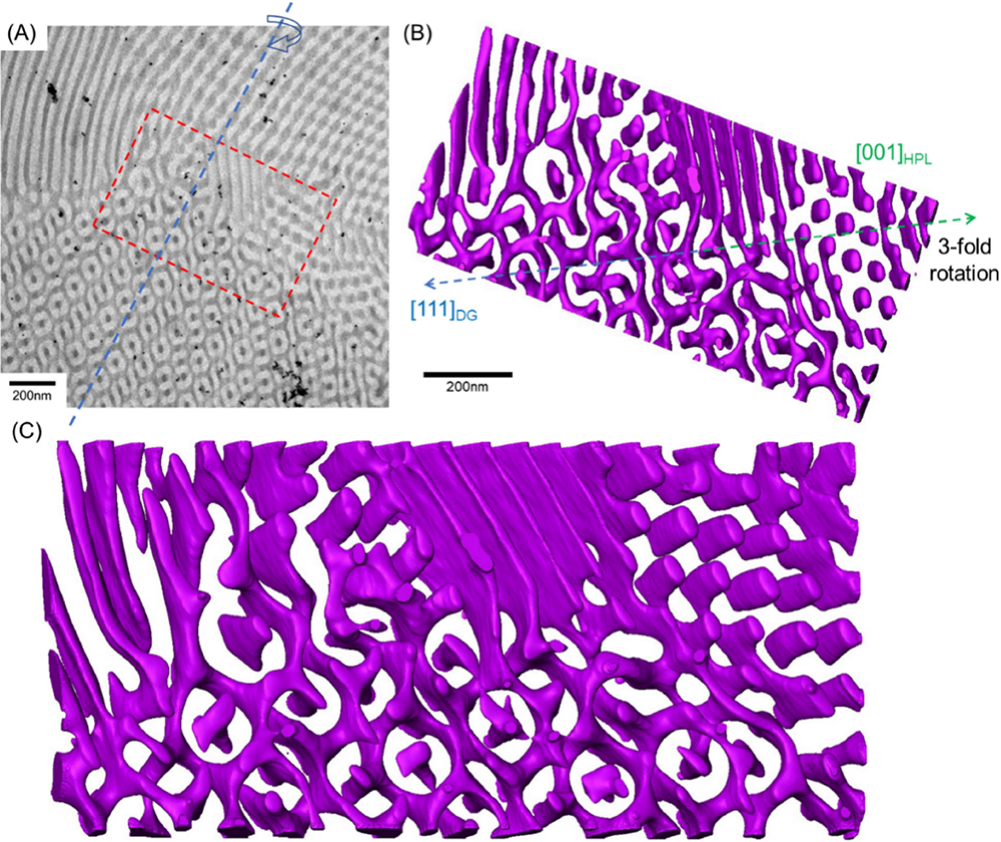
(A) TEM micrograph showing the coexistence of HPL and DG phases from self-assembled PS-*b*-PDMS/PDMS blends with the 3 wt % PDMS homopolymer after solution-casting at a moderate evaporation rate (0.1 mL/day). The structures of PDMS microdomains are three-dimensionally reconstructed from the marked area in (A) and presented as a purple framework along different viewing angles in (B) and (C).

A hypothesis for the mechanism of the phase transition from the HPL to the DG phase is therefore proposed. There may be an adjustment in the geometric dimensions to justify the corresponding lattice mismatch between the HPL and DG phases; in other words, the phase transitions might undergo soft epitaxial growth during reticulation.^45,51,52^ Hence, the solution-cast sample with coexisting phases (i.e., moderate evaporation) is expected to be the critical transition stage for deciphering the epitaxial relationship. Note that the interdomain spacing (*d*) values of (211)_DG_ (i.e., *d*_(211)DG_) and (001)_HPL_ (i.e., *d*_(001)HPL_) were calculated to be 71.6 and 70.0 nm, respectively, based on the primary reflections from the coexistent phases (Figure 4A); similar correspondence in the matching of the two spacings in the HPL/DG mixed phases have been reported; it is believed that the *d*_(211)DG_ and *d*_(001)HPL_ values will reasonably correspond to the coplanes with lattice space matching (i.e., *d*_(211)DG_ ∼ *d*_(001)HPL_) for phase transitions.^42,43^ A suggested epitaxial relationship between the HPL and DG phases is along two parallel [001]_HPL_ and [211]_DG_ directions; note that there is an obvious mismatch between the hexagonal lattice of perforated PS cylinders and the interdomain spacing of (211)_DG_. In the thin-film state, the soft epitaxy between (001)_HPL_ and (211)_DG_ planes has been clearly identified and reported by Chang, Jinnai and co-workers.^42,43^ Interestingly, it is noted that *d*_(100)HPL_ also shows a spacing (∼7% difference) similar to the calculated interdomain spacing for (111)_DG_ which is a forbidden reflection. In addition to the suggested soft epitaxy, there is the question of whether it is possible to have short columns on the perforated layers (PS microdomains) where the perforation cylindrical axes along [001]_HPL_ will transform into a (111)_DG_ matrix. Although SAXS is a powerful tool to derive structural information averaged over a large sample volume, it is incompetent to provide detailed structural information. As a result, direct TEM imaging focusing on the transition zones can be carried out. As shown in Figure 4B,C, projections of the boundaries between the HPL and DG phases from different locations can be clearly identified, further evidencing the coexistence of the mixed phase, as found from the SAXS results. More micrographs of the HPL and DG phase boundaries are included in Figure S9. Electron tomography was thus carried out to visualize the morphological evolution, in particular, the transition zones at the interface. As shown in Figure 6, a reconstructed model from the specific area marked by the red dashed line in the 2D TEM image (Figure 6A) indicates the coexistence of HPL and DG phases with the suggested coplane (a typical character of an epitaxial relationship). Note that the small isolated black particles that can be found in Figure 6A are gold fiducial markers for reconstruction. As shown in Figure 6B,C, based on the results of the reconstructed models, the transition might entail a deformation process from a hexagonal lattice, HPL, to a cubic lattice, DG, at which the isolated PDMS layers would penetrate through the PS matrix, and thereby build connections to each other, leading to the formation of continuous networks from the side of the HPL phase. Subsequently, an epitaxial relationship between the HPL phase along the [001]_HPL_ direction and the DG phase along the [111]_DG_ direction can be identified where the 3-fold rotational symmetries can be identified along the axis (see Figure S10 and Movie S1 for more viewing angles of the region of interest). Note that these results are in line with the observations presented by Hasegawa and co-workers,^53^ who found an epitaxial relationship between HPL layers and the (111)_DG_ gyroid plane as they share the same 3-fold rotational symmetry. Furthermore, as shown in Figure 6C, in place of typical trigonal nodes, the DG phase forming at the interface undergoes a transitional stage that results in a deformed network structure; specifically, the curvatures of the DG network vary significantly, where curved short walls can be spotted at certain locations. In other words, there are no clear-cut boundaries between two phases during the transition. Instead, intermediate regions exist to adjust the symmetry variation between the HPL and DG phases with a soft epitaxial relationship. Accordingly, these results indicate a mechanism similar to the hypothesis proposed previously at which the transition should cause an adjustment in the geometric dimensions, forming the deformed network structures as the embryo of the DG phase with a relatively larger interdomain spacing, while the structures might gradually shrink to a smaller size and turn into the typical trigonal planar to alleviate packing frustration for adjustment of the symmetry variation even with a lower degree of mismatch. As a result, the discoveries of the intermediate region obtained from moderate evaporation reveal how the transformation from isolated and perforated layers to dispersed but continuous network microdomains through symmetry operation can be achieved by rearrangement and justification of microphase-separated domains to fulfill the criteria of thermodynamic stability with respect to packing frustration. Accordingly, these results from in situ and ex situ SAXS experiments, as well as 3D tomography, reveal the possible pathways for the order–order transition from perforation to reticulation, yielding the network structure of DG. Note that the suggested soft epitaxy with the coplanes of (001)_HPL_ and (111)_DG_ is different from the one with the coplanes of (001)_HPL_ and (211)_DG;_ we speculate that the discrepancies might be attributed to the additive effects on the formation of the coplanes in the film state such as thickness commensuration, surface, and substrate for the self-assembled morphologies.

### Order–Order Phase Transition from HPL to DD

**Figure 7:**
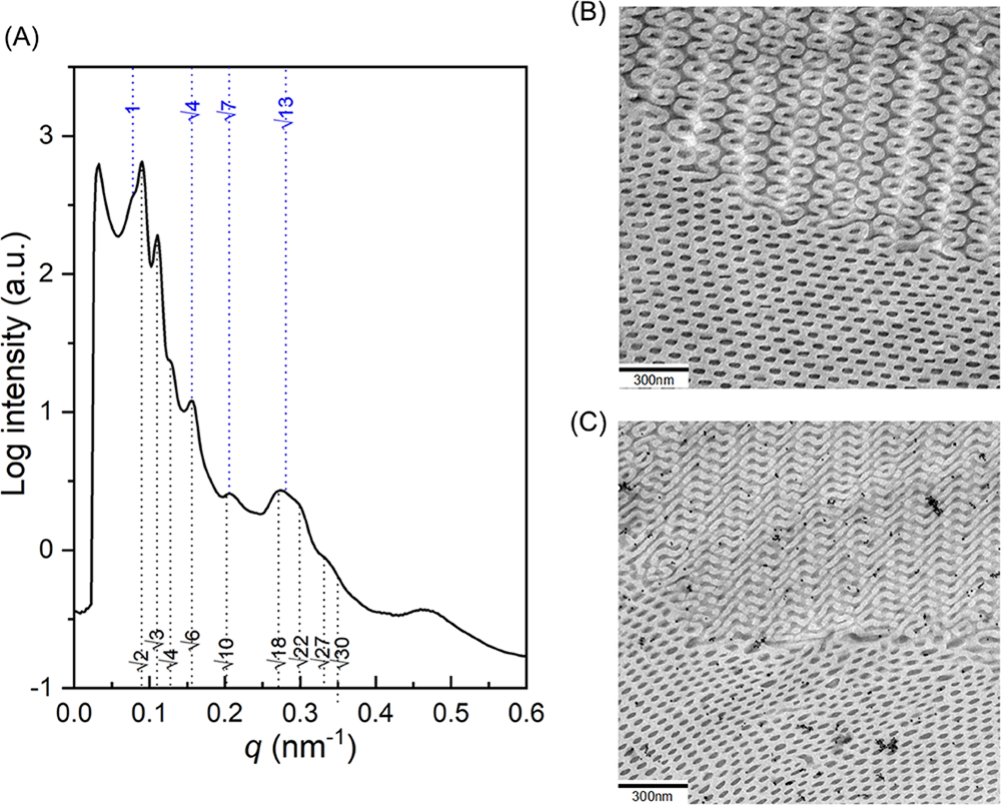
(A) 1D SAXS profile of the solution-cast PS-*b*-PDMS/PDMS blends with 1.5 wt % PDMS homopolymer after moderate evaporation (0.1 mL/day). The blue dashed lines denote the reflections corresponding to HPL, whereas the black dashed lines represent reflections from DD. (B, C) Corresponding TEM micrographs of the boundaries between the HPL and DD phases from different locations.

As discussed previously, there is a consensus that the phase transition between the HPL and DG phases is expectable due to the three-to-three-fold symmetry transformation.^53^ In contrast, the transition between HPL and DD phases might be accompanied by a symmetry-breaking transformation from a trigonal node to a tetrapod, which obviously disobeys a typical epitaxial growth procedure along the trigonal nodes. The self-assembled PS-*b*-PDMS/PDMS blends (1.5 wt % PDMS homopolymer) acquired from solution-casting under a moderate evaporation rate (0.1 mL/day) were prepared for capturing the transition between HPL and DD phases. As shown in Figure 7A, reflections from the SAXS profile evidence the formation of a mixed phase of HPL and DD phases. The blue dashed lines with relative *q* values occurring at 1:√3:2:3:4 suggest the hexagonally packed PS perforation from the HPL phase and the black dashed lines with relative *q* values of √2: √3: √4: √6: √8: √12: √14: √22: √26: √50 indicate the formation of the DD phase. Note that lamellar reflections from the HPL phase can be barely identified from the SAXS profile (Figure 7A); we speculate that the layers from the HPL phase might mostly be deformed and further create the initial stage for the formation of the DD phase (see below for details). Likewise, Figure S11 shows resemblance between the HPL/DD and HPL/DG mixed phases. The interdomain spacing of (110)_DD_ (i.e., *d*_(110)DD_) was determined as approximately 79.4 nm at a moderate evaporation rate and was found to be far larger than the interdomain spacing from slow evaporation (∼66.4 nm), whereas the interdomain spacing of (100)_HPL_ (i.e., *d*_(100)HPL_) was calculated as approximately 72.1 nm, which reduces its size as compared to the one obtained under fast evaporation of the solvent. As a result, it is reasonable to expect that it might require a similar adjustment in the geometric dimensions with a soft epitaxial relationship during the transformation from the HPL to DD phases. Furthermore, as shown in Figure 7B,C, real-spacing images of the boundaries between two phases at different locations can be examined by TEM, giving cogent evidence for the formation of mixed phases of the HPL and DD phases.

**Figure 8:**
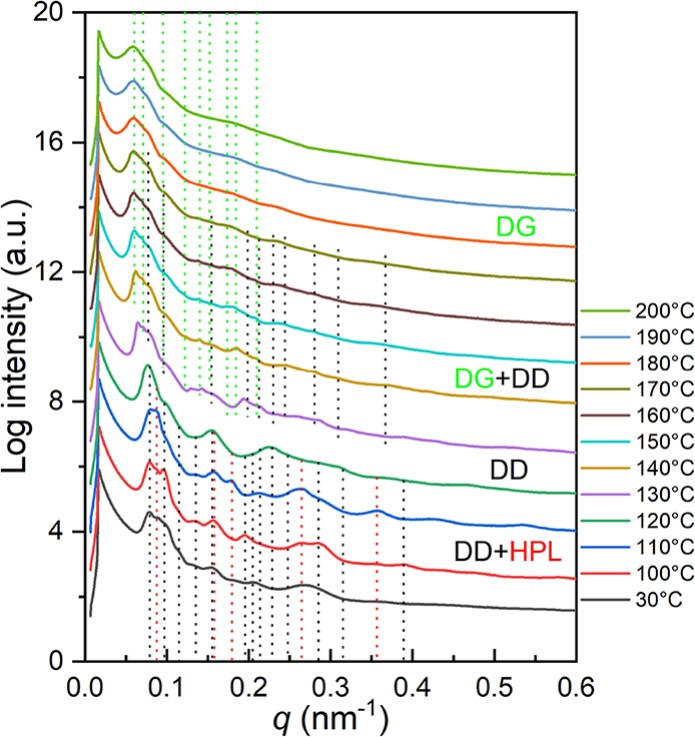
Temperature-resolved in situ 1D SAXS profiles of PS-*b*-PDMS/PDMS blends with the 3 wt % PDMS homopolymer after moderate evaporation (0.1 mL/day) for solution-casting. The black dashed lines denote the reflection planes of a DD phase; red dashed lines denote those of the HPL phase. Green dashed lines represent the reflection planes from the DG phase. The intensities of the scattering profiles are shifted vertically by an arbitrary factor to avoid overlapping of peaks.

As shown in Figure 8, characteristic reflections from both HPL (red dashed lines) and DD phases (black dashed lines) can be clearly identified. In the first stage (<130 °C), similarly, the increase in the intensities of all of the reflection peaks from both HPL and DD phases can be found due to the ongoing thermal annealing that gives rise to long-range ordering as the PS matrix devitrifies. Once the temperature reaches 120 °C, reflections from (100)_HPL_ and (200)_HPL_ decay, whereas the reflections for the DD phases remain, indicating the occurrence of order–order transitions from mixed phases into a single phase (DD phase). When the temperature increases over 130 °C, the reflections from the DD phase broaden; meanwhile, a new peak arises left to the reflection of (110)_DD_. Specifically, a new set of peaks (green dashed lines), which correspond to the characteristic reflections of the DG phase, appear as the temperature increases to 140 °C, suggesting the ongoing order–order transition from the DD phase to the DG phase, as reported previously.^45^ The characteristic reflection planes for the DG are labeled with green dashed lines at relative *q* values of √6: √8: √14: √24: √30: √38: √48. Interestingly, the reflections for the DD phase last for a long period until the surroundings are heated to approximately 180 °C, where no trace of the DD phase can be identified thereafter. Similarly, the completion of phase transformation results in a significant shifting of reflections to a lower *q* region where the interdomain spacing of (211)_DG_ (108.1 nm) will be larger than the interdomain spacing of (110)_DD_ (82.0 nm). The observed sequential step-changing phase transition from HPL to DD, and eventually to the DG phase, supports the hypothetical speculation that the HPL phase should be a metastable phase with lower thermodynamic stability than the other two network phases.

**Figure 9:**
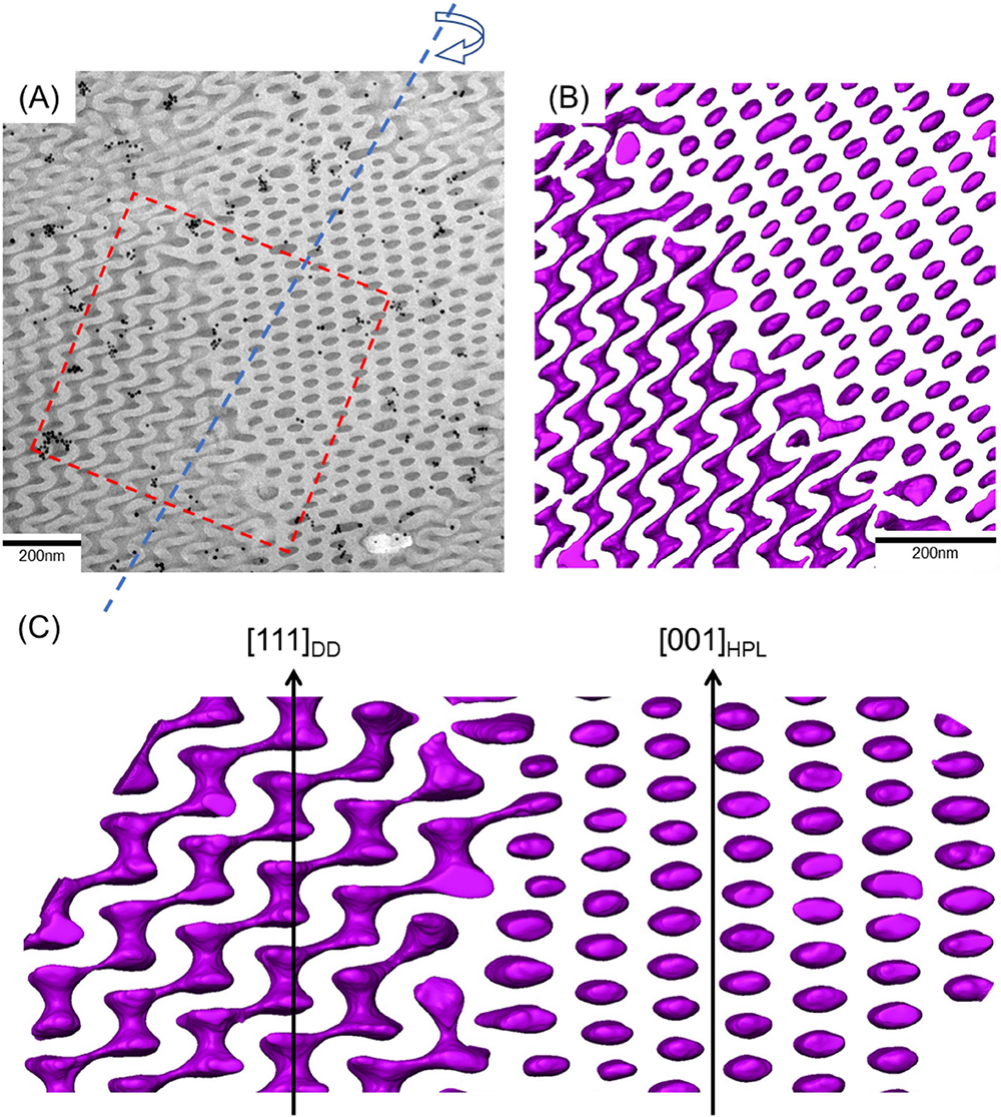
(A) TEM micrograph showing the coexistence of the HPL and DD phases from self-assembled PS-*b*-PDMS/PDMS blends after solution-casting at a moderate evaporation rate (0.1 mL/day). The structures of PDMS microdomains are three-dimensionally reconstructed from the marked area in (A) and presented as a purple framework along different viewing angles in (B) and (C).

**Figure 10:**
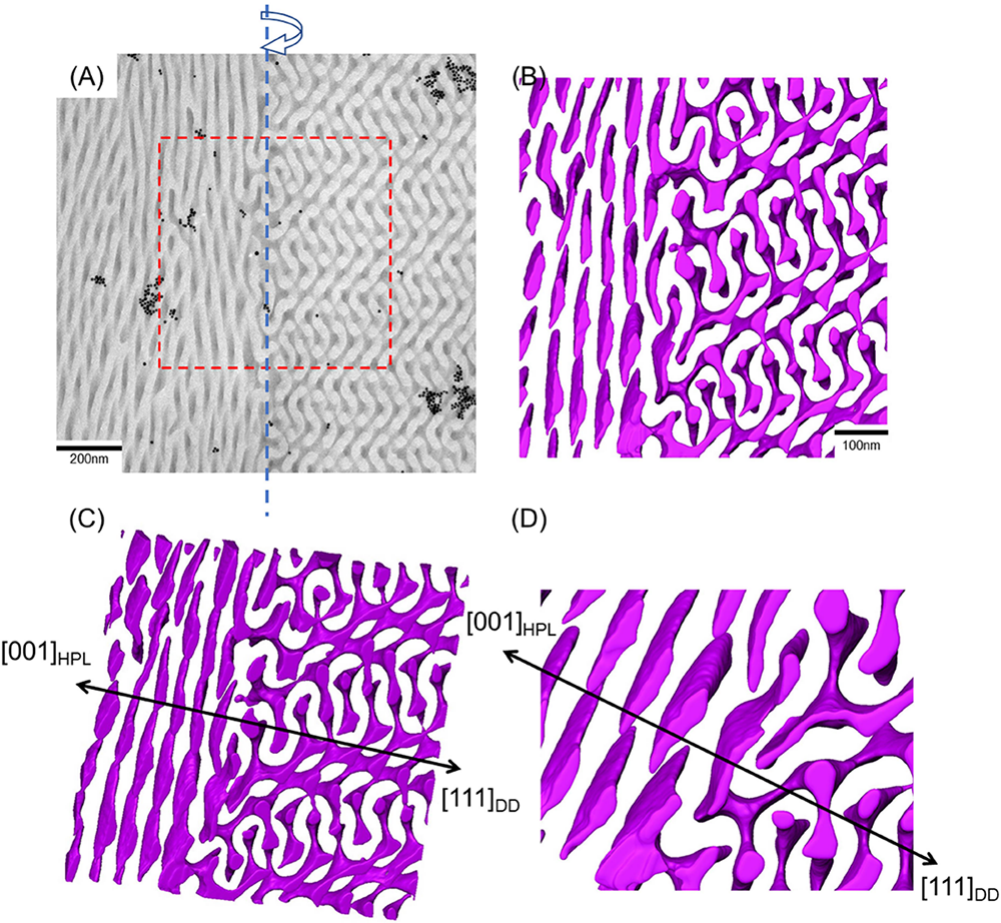
(A) TEM micrograph showing the coexistence of HPL and DD phases at different location from self-assembled PS-*b*-PDMS/PDMS blends after solution-casting at a moderate evaporation rate (0.1 mL/day). The structures of PDMS microdomains are three-dimensionally reconstructed from the marked area in (A) and presented as a purple framework along different viewing angles in (B), (C), and (D).

To study the details of the mechanism of the HPL–DD transition, a specific area marked by a red dashed line in Figure 9A was selected for three-dimensional reconstruction by electron tomography (see Movie S2 for more viewing angles). As shown in Figure 9B, the transition underwent a “matching and connecting” process where isolated short columns were connected to their neighbors, and thus two independent continuous networks with four struts formed. Similarities to the transition between HPL and DG phases can be found except for the alternation on the coordination numbers and strut numbers. Moreover, an interesting transition stage can be identified, where the [001]_HPL_ and [111]_DD_ zonal axes are parallel, as shown in Figure 9C. Obviously, the epitaxial relationship between the HPL phase and DD should be different from the HPL to DG transition where the DG phase is along [111]_DG_. To systematically investigate the epitaxial relationship, different locations showing the boundaries of the mixed phase were captured (Figure 10A and Movie S3). Likewise, as shown in Figure 10B–D, a reconstructed model by electron tomography at a specific area marked by the red dashed line in Figure 10A was also established. Note that both reconstructed models from two different locations (Figures 9 and 10) show distorted perforated PDMS layers, which can barely be distinguished as the ABC-type stacking sequence at the transition zone; we speculate that this might be attributed to the large lattice mismatch between two phases (Figure 7A). Therefore, the disconnectedly compressed columns in different layers of the HPL structure will be significantly deformed while columns are building connection to each other to form network with tetrapod nodes. Meanwhile, the severe deformation of the perforated layers might be the main reason for the lack of lamellar reflections in the SAXS profile (Figure 7A). Furthermore, as shown in Figure 10C,D, an epitaxial growth from the HPL phase along the [001]_HPL_ direction to the DD phase along compressed columns in different layers of the HPL structure will be significantly deformed while columns are building connections with each other to form a network with tetrapod nodes. The [111]_DD_ direction can also be identified, where the network grows from the top of the perforated structure. Similar results are found from other regions showing gradual HPL–DD transition, where the epitaxial relationship of the two connected phases shares the same rotational axis (Figure S12 and Movie S4). However, the target area displays perforated structures with a high density of dislocation defects and distortion, which may be attributed to the formation of intermediate states before transformation into DD phase. As a result, on the basis of these reconstructed models (Figures 9 and 10), it is interesting to conclude that the DD phase can grow from the side or the top of the HPL phase; in addition, they both indicate that the nucleation process is initiated from the (001)_HPL_ plane (i.e., hexagonally packed cylindrical central axis), and the growth of the DD phase will be followed by [111]_DD_. Accordingly, the transition leads the corresponding geometry of (001)_HPL_ to (111)_DD_ while they might share the same 3-fold rotational and mirror symmetry, causing a possible transition through a three-to-three symmetry transformation in two-dimensional space along [001]_HPL_ and [111]_DD_ directions from the HPL to the DD phase. It is worthy noticing that the transition leads to the formation of the tetragonal network structure from both the top and the side of the HPL phase with the suggested coplane. Furthermore, focusing on the boundary between the HPL and DD phases (Figure 10D), the forming, extruded tetrapod nodes from isolated elliptical layers can be clearly observed, indicating the emergence of the intermediate stage in the mixed phase. As a result, the intermediate region can be profoundly found during the transition from the HPL to the DD phase due to the necessity of space for lattice adjustment and also the restricted symmetry operation during phase transition to the intrinsic DD phase.

As a result, owing to the geometric confinement in two-dimensional space for matching HPL phase (i.e., 3-fold symmetry), a specific plane (i.e., (111)_DD_) with 3-fold symmetry from the DD phase reasonably shares the coplane with (001)_HPL_ from the HPL phase, although there is a significant lattice mismatch between the two planes. Therefore, to alleviate the lattice mismatch problems, the structure is quite disorganized at the boundary of both perforated layers from the HPL phase and the initial stage for the formation of the DD phase. On the other hand, a three-to-three-fold symmetry transition could also occur during the transition from a HPL phase to a DG phase, as discovered by Hasegawa and co-workers^53^; however, the torsion angle of the trigonal planar network and chirality of the gyroid structure suggest the twisting of the perforated structure with 3-fold symmetry and the lattices match along the [111]_DG_ and [001]_HPL_ directions,^14,54^ which is beyond the “nucleation process” observed from the HPL to DD transition. Conclusively, both transformations undergo intermediate stages (i.e., deformed network structures) during the evaporation for the formation of intrinsic network structures from perforations, which means that polymer chains, which serve as “soft” matters, are able to rearrange and adjust to different lattices in the geometric dimensions for reticulation and further give rise to a “soft” epitaxial relationship between perforation and network structures.

## Conclusions

In conclusion, diverse morphologies can be kinetically trapped from lamellae-forming PS-*b*-PDMS BCPs by controlled self-assembly and the introduction of a low-molecular-weight PDMS homopolymer. An HPL phase was identified to exist as an intermediate metastable phase during the morphological evolution from the network phases as evidenced by real-/reciprocal-space images. Interestingly, the perforation from HPL in ABC-type stacking (*R*3̅*m*) was discovered to be an initial stage for reticulation to three-dimensional network structures, such as gyroid (DG) and diamond (DD) phases. By taking advantage of electron tomography and SAXS examinations, epitaxial growth from the [001]_HPL_ to [111]_DG_ directions can be clearly observed. Interestingly, the finding is in line with the results from previous reports, which revealed an epitaxial relationship as they share the same three-to-three-fold rotational symmetry. On the other hand, a similar epitaxial relationship can be identified from the transition between the HPL and DD phases but along the [001]_HPL_ and [111]_DD_ directions. The specific plane (i.e., (111)_DD_) from the DD phase exhibits a possible transition through a 3-fold symmetry in two-dimensional space to match a 3-fold-symmetry HPL phase and further shares the coplane with (001)_HPL_. As a result, according to these discoveries, “soft” epitaxial relationship can be interestingly identified within the transition from perforation to reticulation for BCPs.

## Supporting Information Available

Details of sample preparation and morphological observation of the PS-*b*-PDMS and PS-*b*-PDMS/PDMS blends through controlled self-assembly (PDF)
Animation of the three-dimensional structures of PDMS microdomains reconstructed from the marked area in Figure 6A (MOV)
Animation of the three-dimensional structures of PDMS microdomains reconstructed from the marked area in Figure 9A (MOV)
Animation of the three-dimensional structures of PDMS microdomains reconstructed from the marked area in Figure 10A (MOV)
Animation of the three-dimensional structures of PDMS microdomains reconstructed from the marked area in Figure S12A (MOV)

## Supplementary Material

am4c20386_si_001.pdf

am4c20386_si_002.mov

am4c20386_si_003.mov

am4c20386_si_004.mov

am4c20386_si_005.mov
