# Supplementary material for: Reticulation of Block Copolymer Nanostructures from Perforation
Source: ACS Appl Mater Interfaces. 2025 Feb 12;17(8):12676–85. doi: 10.1021/acsami.4c20386 (PMC11873956; doi:10.1021/acsami.4c20386)
Supplement: Supplementary file 1 — am4c20386_si_001.pdf [file am4c20386_si_001.pdf]

Supporting Information for

Reticulation of Block Copolymer Nanostructures from Perforation

Shih-Lin Yeh [†,‡], *Cheng-Yen Chang,* [†,‡] *and Rong-Ming Ho*[*,†]

[†] Department of Chemical Engineering, National Tsing Hua University, Hsinchu 30013, Taiwan, R.O.C.

[‡] S.-L. Yeh and C.-Y. Chang contributed equally.

* To whom correspondence should be addressed.
Tel: 886-3-5738349; Fax: 886-3-5715408; E-mail: rmho@mx.nthu.edu.tw
Department of Chemical Engineering, National Tsing Hua University, Hsinchu 30013, Taiwan, R.O.C.

Table of Contents

Materials.....3

Sample Preparation.....5

Morphological Observation.....6

Controlled Self-Assembly of PS-*b*-PDMS and PS-*b*-PDMS/PDMS Blends.....8

Other Supporting Materials.....13

References.....13

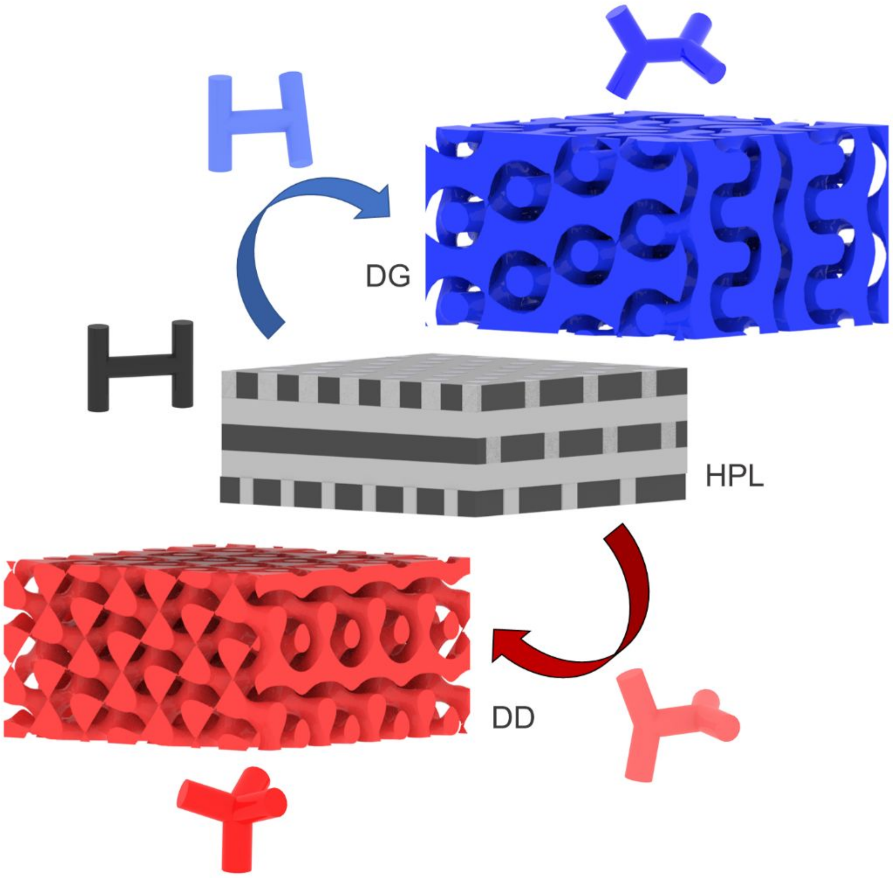

Figure S1. Schematic illustration of the hypothetical order-order transitions from perforation to reticulation among the HPL, DG and DD phases.

Materials

The lamellae-forming PS-*b*-PDMS with f_{PDMS}^{v} at approximately 0.42 used for investigation of kinetically captured complex in this study has been reported previously.[1] Detailed characterization, including the size exclusion chromatograph (SEC) and the proton nuclear magnetic resonance (^{1}H-NMR) spectrum, of the studied diblock PS-*b*-PDMS were exhibited in **Figure S2**. More details of the molecular characterization were summarized in **Table S1**. Commercial PDMS-OH purchased from *Polymer Source Inc.* was used in this study. As illustrated in **Figure S3,** ^{1}H-NMR ($CDCl_3$): 5.05 (m, 1H, CH), 3.51 (d, 2H, CH_2), 1.52 (br, 2H, CH_2), 1.48 (br, 2H, CH_2), 1.35 (br, 3H, CH_2), 0.89 (t, 3H, CH_3), 0.52 (t, 3H, CH_2), 0.14-0.34 (m, 6H, $Si(CH_3)_2$) can be clearly identified, which is in line with the chemical structure of commercial PDMS-OH**.** The peak area ratio of peak h (repeating unit) and peak g (n-butyl end group) could be calculated to acquire the molecular weight of PDMS-OH. On the basis of the area ratio calculation, the molecular weight of commercial PDMS-OH was determined as approximately 10,000 g/mol. As shown in **Figure S4**, the GPC result of the PDMS-OH gives a clear major peak with a minor shoulder peak, suggesting that there should be small amount of high molecular weight while the major part of the PDMS-OH indeed reflects a sharp peak. We speculate that there might be the inhomogeneity of the PDMS-OH purchased, resulting in the dual population. Yet, the polydispersity of the commercial PDMS-OH is approximately 1.07 as determined; it might be still available to be used as a reasonably good wet-brush like texture due to its narrow polydispersity.

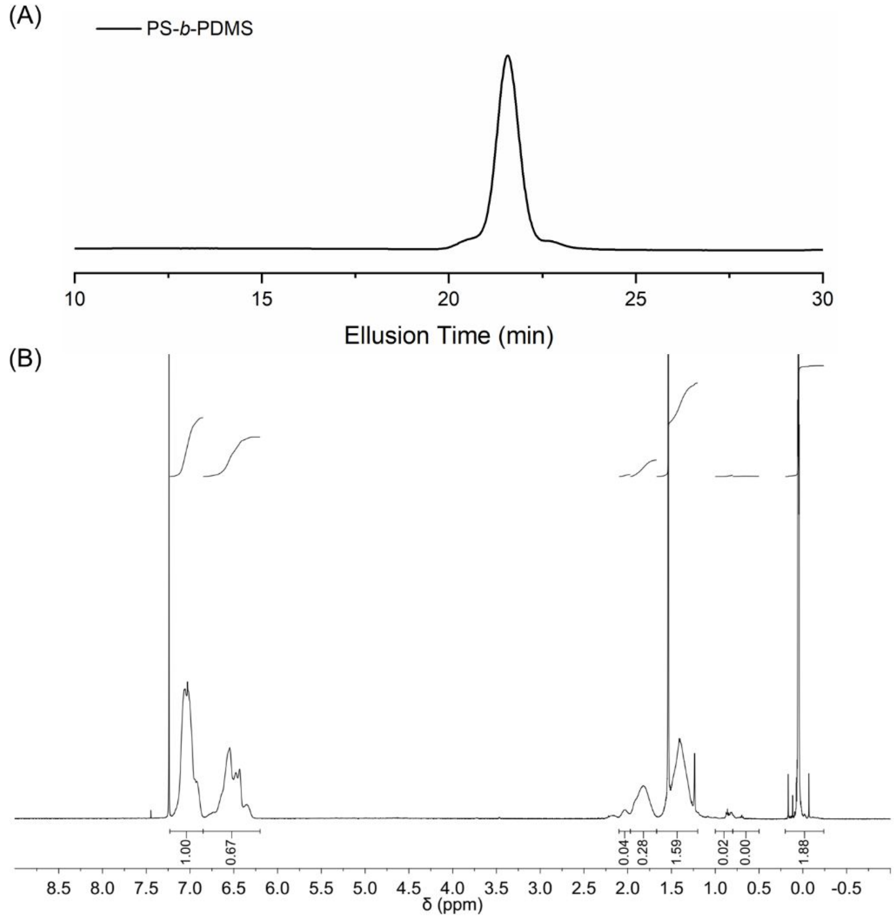

Figure S2. (A) The chromatograph and (B) the ^{1}H NMR spectrum of PS-*b*-PDMS.

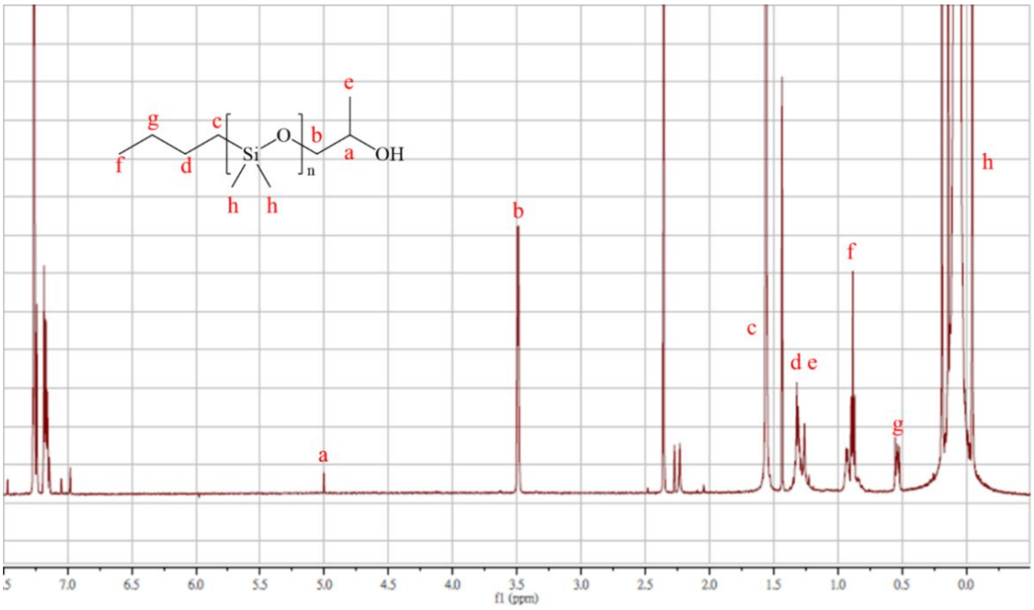

Figure S3. The ^{1}H NMR spectrum of the commercial PDMS-OH.

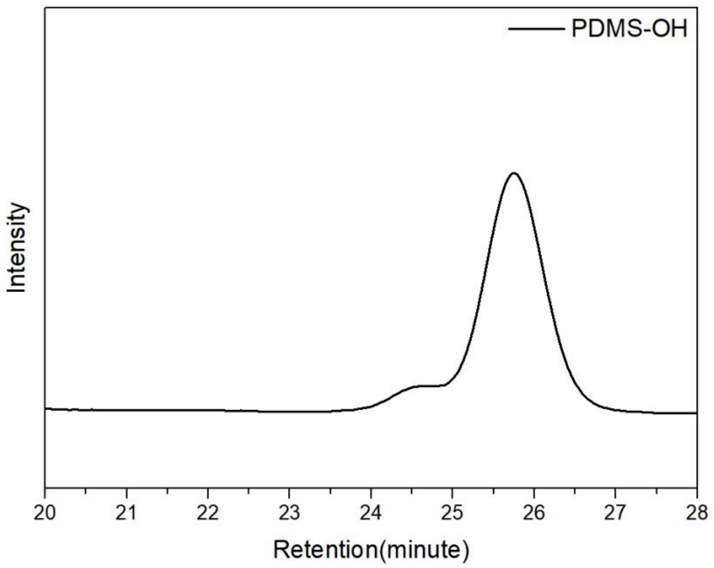

Figure S4. The SEC chromatograph of the commercial PDMS-OH.

Table S1. Typical characterizations of the lamellae-forming PS-*b*-PDMS and PDMS-OH.

Sample	$\overline{M}_n^{PS}$ kg/mol [a]	$\overline{M}_n^{PDMS}$ kg/mol [a]	$\overline{M}_n^{total}$ kg/mol [b]	$Đ_M$ [c]	f_{PDMS}^{v} [d]
PS-*b*-PDMS	51.0	35.0	86.0	1.05	0.42
PDMS-OH	-	10.0*	10.0	1.07	1

[a]Total number-average molecular weights corresponding to two individual blocks (PS and PDMS) determined by vapor pressure and membrane osmometry (VPO and MO). *For the PDMS homopolymer, only VPO was used for measurement.
[b]Total number-average molecular weight of the copolymers measured by VPO and MO.
[c]Polydispersity determined by size exclusion chromatography (SEC).
[d]Volume fraction of PDMS as calculated from proton nuclear magnetic resonance spectroscopy (^{1}H-NMR) (ρ_{PS} = 1.04 g cm^{-3}, ρ_{PDMS} = 0.97 g cm^{-3}).

Sample Preparation

All bulk samples of PS-*b*-PDMS and PS-*b*-PDMS/PDMS blends were prepared by the solution casting at ambient condition using a chloroform (PS-selective solvent) or cyclohexane (C_6H_{12}) which can be referred as a neutral solvent for both PS and PDMS segments. The added amounts of the PDMS homopolymers are fixed at 1.5 wt%, 3wt% and 5 wt% to the weight of the PS-*b*-PDMS. The concentration of polymer solution was fixed at 10 wt %. The initial amounts of polymer solution were all fixed at approximately 500 mg and kept in cylindrical

glass vials (inner diameter ~ 7 mm). Different numbers of pinholes with uniform diameter (~ 600 μm) were punctured on the vial caps for control of evaporation rates. Two evaporation rates of solvent were applied to avoid of the formation of kinetically trapped morphologies: 0.04 and 1.2 ml/day. Subsequently, all bulk samples were detached from glass vials and then transferred to a vacuum oven for 1 day for removal of residual solvent.

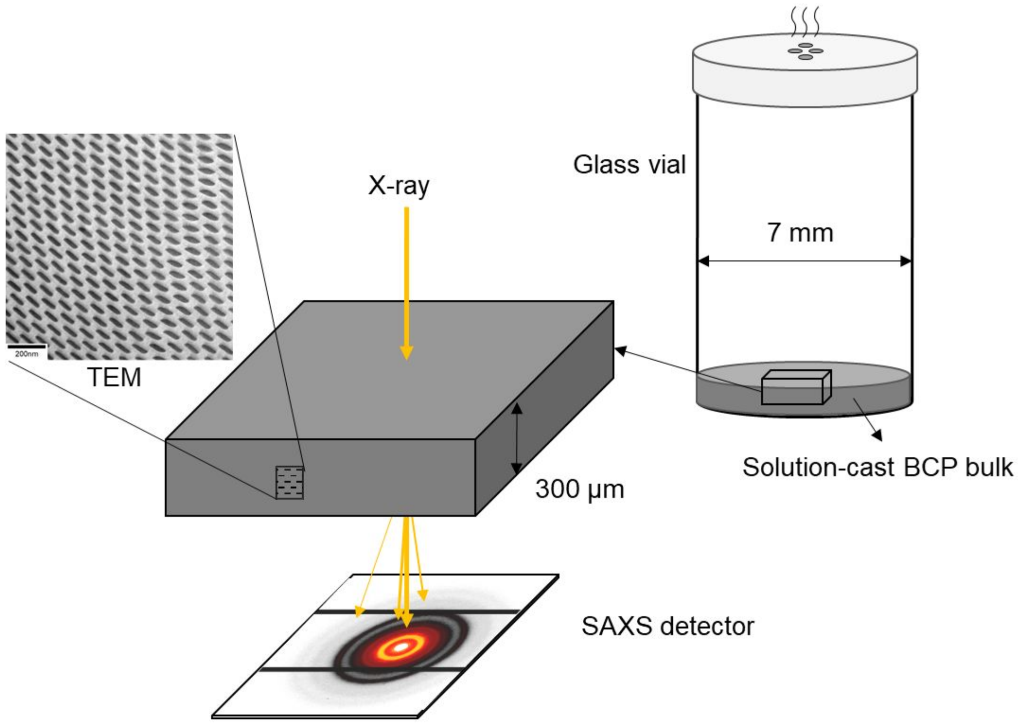

Figure S5. Schematic illustration of the experimental setups for sample preparation and the following morphological observation examined by TEM and SAXS.

Morphological Observation

Ultrathin microsections (thickness lower than 100 nm) of the solution-cast PS-*b*-PDMS and PS-*b*-PDMS/PDMS blends were prepared at -160℃ by a Leica EM UC6 microtome with accessory for cryo-microtome (Cryochamber EM FC7). Real-spacing images (TEM) were acquired from the ultrathin microsections without staining due to intrinsic mass-thickness contrast from PDMS to PS microdomains. TEM studies were performed on a JEOL-2100 Transmission electron microscopy (TEM) operating at an accelerating voltage of 200 kV.

For further examination of the intrinsic phase behaviors of PS-*b*-PDMS and PS-*b*-PDMS/PDMS blends synthesized, all the casted samples were prepared for real-space observation under TEM. As shown in **Figure S6A**, the alternating dark and bright stripes evidenced the formation of lamellar morphology, well consistent with the obtained scattering profiles shown in **Figure S6B**.

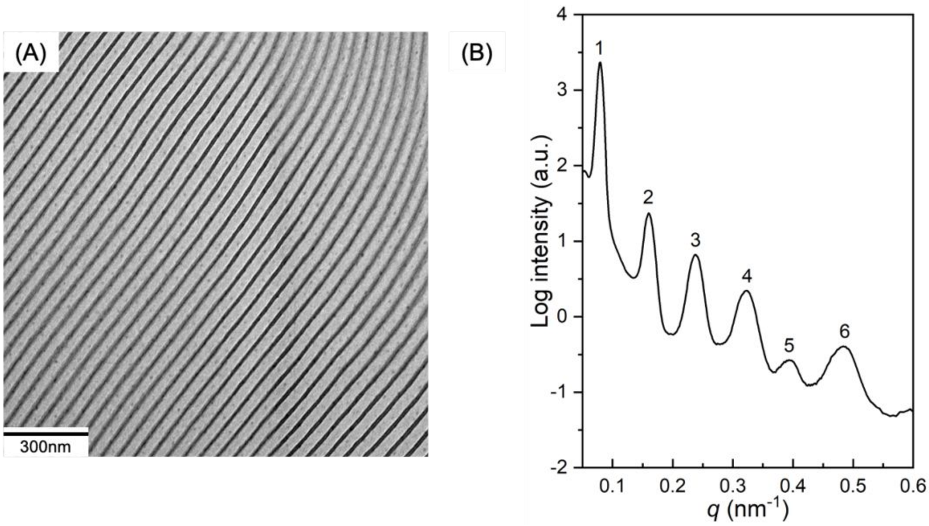

Figure S6. (A) TEM micrograph and (B) 1D SAXS profile of solution-cast PS-*b*-PDMS after thermal annealing at 180 °C for 12 hours.

Controlled Self-Assembly of PS-*b*-PDMS and PS-*b*-PDMS/PDMS Blends.

Table S2. Structural Information of the self-assembled PS-*b*-PDMS/PDMS blends prepared by solution casting using chloroform under fast (1.2 ml/day) and slow (0.04 ml/day) evaporation rates.

Fast evaporation (1.2 ml/day)			
	Morphology	Primary reflection (nm^{-1})	Inter-domain spacing (nm)
PS-*b*-PDMS-1.5wt%	HPL	0.082	76.62
PS-*b*-PDMS-3wt%	HPL	0.077	81.60
PS-*b*-PDMS-5wt%	HPL	0.069	91.06
Slow evaporation (0.04 ml/day)			
PS-*b*-PDMS-1.5wt%	DD	0.095	66.14
PS-*b*-PDMS-3wt%	DG	0.097	64.78
PS-*b*-PDMS-5wt%	L	0.081	77.57

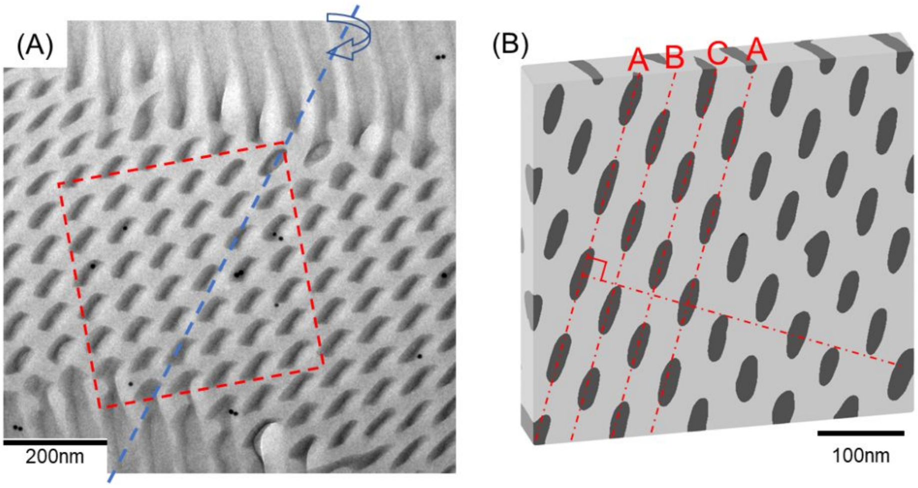

Figure S7. (A) TEM micrograph of self-assembled PS-*b*-PDMS/PDMS blends with 3 wt% of PDMS homopolymer prepared by solution casting using chloroform under fast evaporation rate (1.2 ml/day) and followed by thermal annealing at 120°C for 10 days. (B) Represented three-dimensional model along tilted viewing angle from the marked area in (A) for three-dimensional reconstruction.

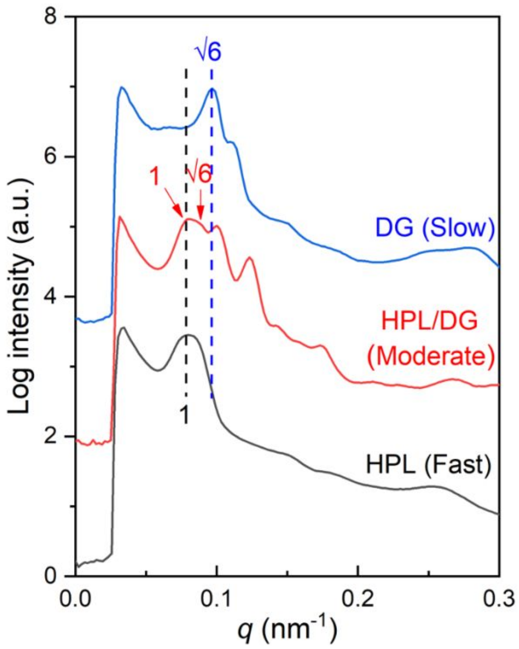

Figure S8. 1D SAXS profiles of solution-cast PS-*b*-PDMS/PDMS blends with 3 wt% of PDMS homopolymer under fast (1.2 ml/day), moderate (0.1 ml/day), and slow evaporation rate (0.04 ml/day) for systematic comparison. The intensities of the scattering profiles are shifted vertically by an arbitrary factor to avoid overlapping of peaks.

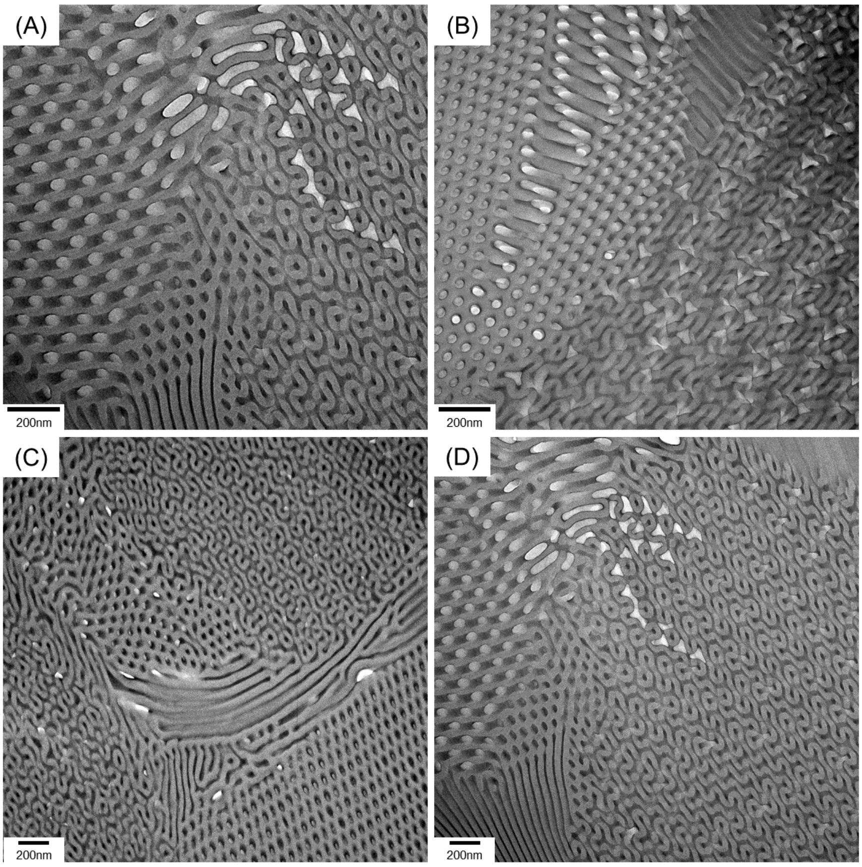

Figure S9. TEM micrographs of the solution-cast PS-*b*-PDMS/PDMS blends with 3 wt% of PDMS homopolymer after moderate evaporation (0.1 ml/day).

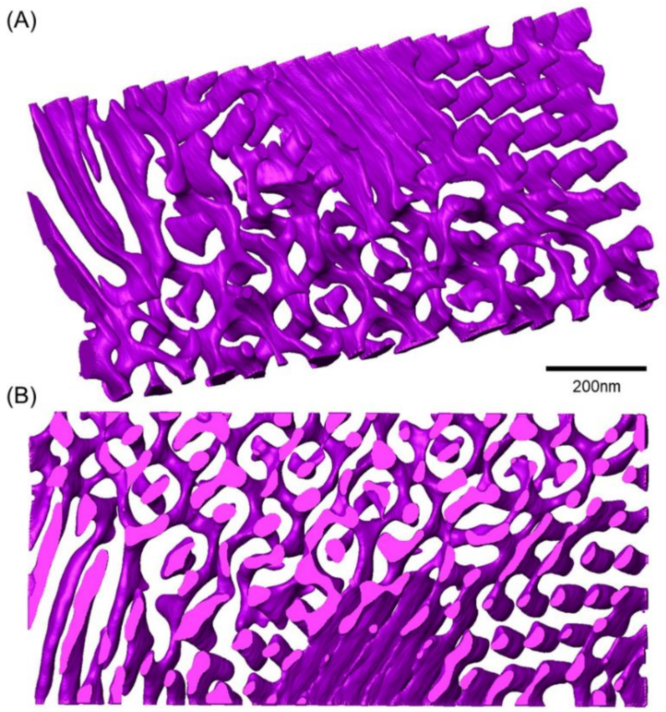

Figure S10. Different viewing angles of the 3D model visualized by reconstruction of the HPL-DG transition zone shown in **Figure 6A**.

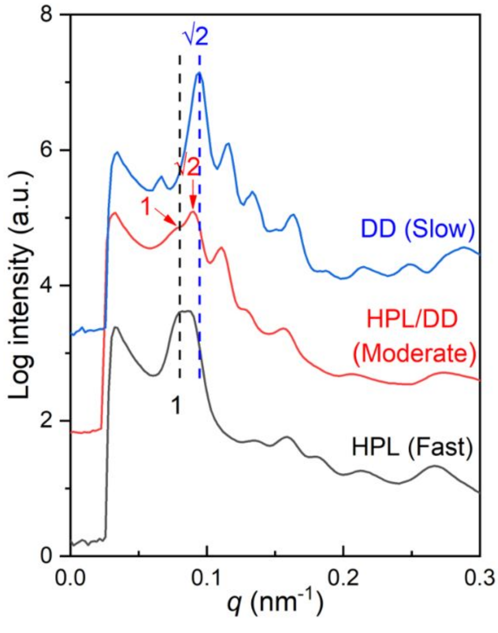

Figure S11. 1D SAXS profiles of self-assembled PS-*b*-PDMS/PDMS blends with 1.5 wt% of PDMS homopolymers prepared by solution casting using chloroform under fast (1.2 ml/day), moderate (0.1 ml/day), and slow evaporation (0.04 ml/day). The intensities of the scattering profiles are shifted vertically by an arbitrary factor to avoid overlapping of peaks.

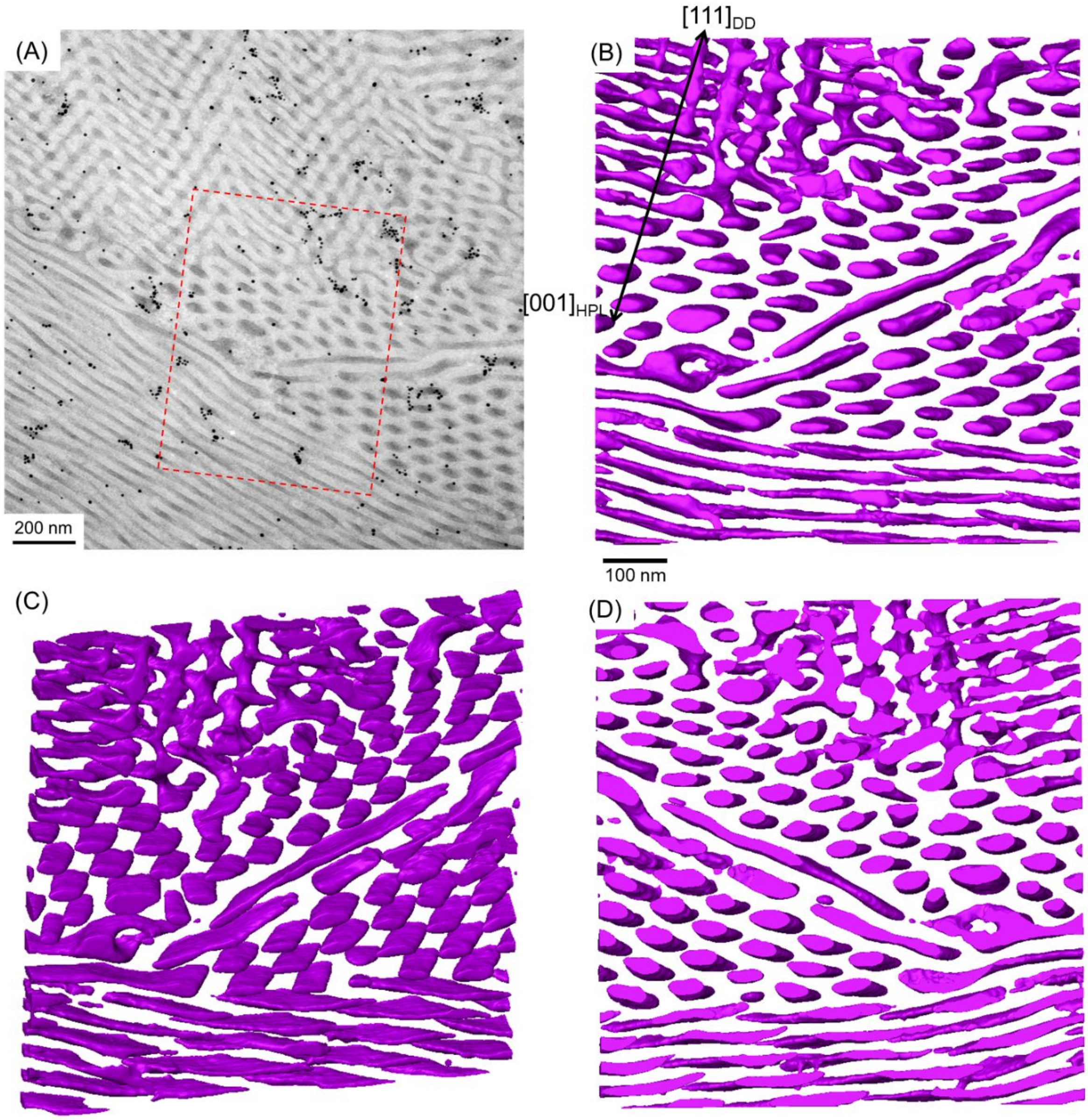

Figure S12. (A) TEM micrograph of the coexistence of HPL and DD phases at different location from self-assembled PS-*b*-PDMS/PDMS blends prepared by solution casting under moderate evaporation rate (0.1 ml/day). The PDMS microdomains were reconstructed from the marked area in (A) and presented as a purple model for demonstrations along different viewing angles in (B-D).

Other Supporting Materials

Movie S1. Animation of the three-dimensional structures of PDMS microdomains reconstructed from the marked area in **Figure 6A**.

Movie S2. Animation of the three-dimensional structures of PDMS microdomains reconstructed from the marked area in **Figure 9A**.

Movie S3. Animation of the three-dimensional structures of PDMS microdomains reconstructed from the marked area in **Figure 10A**.

Movie S4. Animation of the three-dimensional structures of PDMS microdomains reconstructed from the marked area in **Figure S12A**.

References

(1) Chang, C. Y.; Manesi, G. M.; Yang, C. Y.; Hung, Y. C.; Yang, K. C.; Chiu, P. T.; Avgeropoulos, A.; Ho, R. M. Mesoscale networks and corresponding transitions from self-assembly of block copolymers. *Proc Natl Acad Sci U S A* 2021, *118* (11), e2022275118.
